# Low Exercise Capacity Increases the Risk of Low Cognitive Function in Healthy Young Men Born Preterm: A Population-Based Cohort Study

**DOI:** 10.1371/journal.pone.0161314

**Published:** 2016-08-22

**Authors:** Jenny Svedenkrans, Jan Kowalski, Mikael Norman, Kajsa Bohlin

**Affiliations:** 1 Division of Pediatrics, Department of Clinical Science, Intervention and Technology, Karolinska Institutet, Stockholm, Sweden; 2 Department of Neonatology, Karolinska University Hospital, Stockholm, Sweden; University of Pécs Medical School, HUNGARY

## Abstract

**Background:**

Preterm birth is a risk factor for decreased exercise capacity and impaired cognitive functions in later life. The objective of this study was to disentangle the associations between preterm birth, physical fitness and cognitive performance in young adulthood.

**Methods:**

This population-based cohort study included 218,802 young men born in Sweden 1973–1983. Data on birth characteristics was obtained from the Medical Birth Register and linked to exercise capacity assessed by ergometer cycling and cognitive tests performed at conscription for military service in 1993–2001. Cognitive performance was assessed using stanine (STAndard NINE) scores. The results were adjusted for socioeconomic factors.

**Results:**

Exercise capacity was positively associated with cognitive performance across all gestational ages. The sub-group of men who were born extremely preterm (gestational age <28 weeks) and had low exercise capacity exhibited the lowest odds ratio (OR = 0.26, 95%CI:0.09–0.82) of having a cognitive function above the mean stanine score (2.9) for men born at term with normal birth weight. Men born extremely preterm with a high exercise capacity had similar or even higher ORs for cognitive function (OR = 0.59; 95% CI:0.35–0.99) than men born at term with low W_max_ (OR = 0.57; 95% CI:0.55–0.59).

**Conclusions:**

Physical fitness is associated with higher cognitive function at all gestational ages, also in young men born extremely preterm. Targeting early physical exercise may be a possible intervention to enhance cognitive performance and educational achievements in populations at risk, such as childhood and adult survivors of preterm birth.

## Introduction

About 10% of the 135 million babies born every year are delivered preterm (before 37 weeks of gestation) [1, 2]. The survival rates for preterm infants are steadily rising, resulting in growing numbers of children and adults born preterm. Survivors of preterm birth are at risk for a variety of health challenges out of which lower cognitive function and lower academic achievements are among the most well-known [3, 4]. Lower cognitive function in children born preterm may result from specific neonatal morbidities such as brain hemorrhages, white and grey matter damage and inflammatory response to infections [5, 6]. But also healthy survivors of preterm birth may suffer from reduced cortical growth during infancy [7], which may contribute to delayed neurodevelopment.

An important part of cognitive function is brain plasticity, which refers to the ability of the brain to adapt to a new situation or environment. Physical exercise, as indexed by cardiovascular fitness, seems to strongly affect brain plasticity [8]. Cognitive function has been correlated to the level of exercise capacity [9, 10] and exercise has been shown to enhance cognitive function in both animals and humans [10–12]. Besides lower cognitive function, subjects born preterm have a lower exercise capacity in young adulthood than subjects born at term [13, 14].

We aimed to explore any associations between exercise capacity and cognitive performance in healthy young men born at successively lower gestational age. We hypothesized that: (1) low gestational age at birth is associated with lower cognitive function, (2) low exercise capacity is associated with lower cognitive function and (3) that a combination of both would be associated with the poorest cognitive outcome.

## Methods

### Ethics

The study protocol was approved by the regional ethical review board in Stockholm (EPN, Regionala Etikprovningsnamnden, Stockholm). Waived consent was approved by the board and individual consent was not obtained from participants included in the study. The final register-based dataset released to the authors and used for analyses was anonymized.

### Study design

This cohort study was based on data from four population-based Swedish registers; the Conscript Register, the Medical Birth Register (MBR), the Population and Housing Census 1990 and the Multigeneration Register. The national registration number, assigned to each Swedish resident at birth, was used for individual record linkage. Linking of data from the different registers was performed by the Central Bureau of Statistics Sweden.

**Fig 1:**
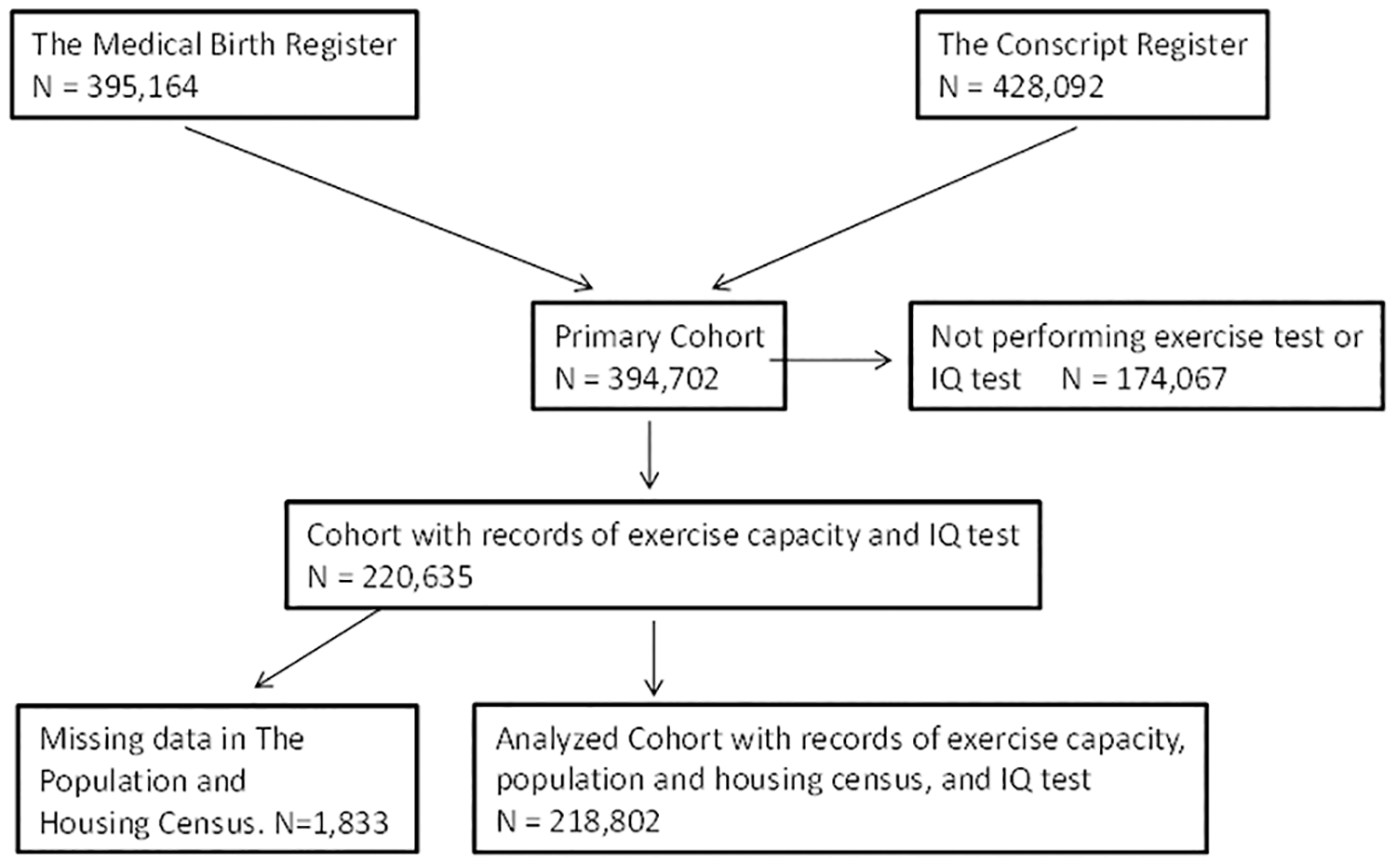
Formation of the study cohort. All men who were registered in both the Medical Birth Register (MBR) and the Conscript Register formed the primary cohort. Men who did not perform the exercise or the IQ test were excluded as well as men with missing data in the Population and Housing Census. Finally, subjects with data in the MBR which were judged as misclassified were excluded.

The Conscript Register contains information about young men assessed for military service. Conscription includes physical examination, health assessment, tests of exercise capacity and cognitive function. The result from the cognitive function test was used as outcome in this study. All men conscripted for military service in 1993–2001 and born in Sweden 1973–1983 were eligible for the study, but subjects without records on perinatal risk factors, exercise capacity or of cognitive function were excluded (Fig 1). Military service is no longer mandatory in Sweden, and therefore the chosen study period corresponds to a time when conscription was enforced by law and included extensive testing. At the time, all Swedish men were drafted, but men with severe handicaps or congenital malformations generally received an exemption. Other reasons for not being conscripted were moving out of the country, death or conscription during another time period.

The MBR contains data on >99% of all births in Sweden. Starting at the first prenatal visit, information is prospectively collected on standardized forms and forwarded to the register. The MBR has been validated, and the quality was considered high [15].

The Population and Housing Census 1990 is a register to which adults in Sweden reported facts about education, profession, household, income and family structure in 1990. It was mandatory for all citizens 16 years and older. The response rate was 97.5% [16].

In The Multigeneration Register, blood relationship of all Swedish citizens is registered. It was used to identify mothers and fathers of the conscripts.

#### Risk factors, confounders and co-variates

Perinatal risk factors were obtained from the MBR. Gestational age (GA) in complete weeks was estimated from the date of the last menstrual period [17]. Subjects with records of GA <22 or >45 weeks were excluded. GA was categorized into 5 groups: extremely preterm (<28 weeks), very preterm (28–31 weeks), moderately preterm (32–36 weeks), term (37–41 weeks) and post term (≥42 weeks). Birth weight (BW) was measured in gram. Values <300 g and >7000 g were judged misclassified and excluded. Birth Weight Standard Deviation Score (BWSDS) was used as a measure for degree of large- and small-for-gestational age. BWSDS was calculated using a Swedish reference for normal fetal growth [18] and BWSDS was divided into five groups: <-2SD, -2SD to <-1SD, -1SD to <+1SD, +1SD to <+2 SD and ≥+2SD. Study subjects with a BWSDS exceeding ±6SD were excluded.

In addition, the following variables from the MBR were obtained: maternal age (<20, 21–25, 26–30, 31–35, 36–39, >40 years), maternal origin of birth (born in Sweden, other Nordic countries, other European countries, Asia or other countries including North America, South America, Africa and Oceania), parity (primi- or multipara) and singleton or multiple birth.

Income of parents, including salary and income from finances, business and land or forest area, was retrieved from the Population and Housing Census 1990. Income was grouped in quartiles, the first quartile was considered as low, second and third as average and fourth quartile as high income. In this study, the highest income of mother or father was used.

The parental educational level of the conscript was reported in 8 levels and defined as the highest of mother or father. Socioeconomic index (SEI) categorizes people according to occupation. There are 15 index categories ranging from leading position, farmers and workers to non-occupied. Each index category has a dominance in relation to the others that is defined based on the expected impact on the child’s SEI [19]. In the study a subject’s SEI was set as the most dominant SEI of the parents.

Exercise capacity was obtained from the Conscript Register and defined as the maximal load (expressed in Watt, W_max_) that the conscript could manage on cycle ergometer test. Only conscripts with a normal electrocardiography and without diseases or injuries that beforehand were known to influence the results were allowed to perform the test. The test was performed according to the conscription protocol with initial workload determined by weight (125 W for weight 70 kg). For men who exercised on a regular basis, the initial work load was increased by 25W. After 5 min cycling on submaximal load with a pulse between 120 and 170, the load was increased by 25 W every minute as long as the conscript managed. The test was ended when the conscript acknowledged that he had reached his maximum capacity or when he stopped cycling for 8 second or more. If the maximum heart rate was lower than 180 the test could be re-done. The conscript was instructed to perform to his maximal capacity. For statistical analysis, the results from the exercise capacity test were block stratified into groups from low to high range (≤225W, 226-275W, 276-325W, 326-375W, >375W) based on the previously known mean level of exercise capacity in the cohort [13].

Body Mass Index (BMI <18, 18–24.9, 25–29.9, 30–35, >35 kg/m^2^) and blood pressure (mmHg)—measured in the right arm after 5 to 10 min rest in the supine position—were collected from the conscript register.

A health assessment was performed by a physician at conscription. Based on physical examination, previous and current medical history, the conscripts were categorized in 8 health levels with A being the highest category. Level A represents fully healthy individuals without any minor health problem. Every lower level adds one or more health problem from mild allergies to asthma and severe diseases (S1 Table).

#### Outcome

Results from the cognitive function test were derived from the Conscript Register. Cognitive function was tested at conscription by a time-limited written test consisting of 160 questions divided equally into four parts evaluating spatial, verbal, logical/inductive and theoretical/technical skills. The overall result for each subject was recalculated into a stanine score from 1–9. A stanine score (STAndard NINE) is a statistical instrument used to scale test results that follow a normal distribution. Average is 5 and standard deviation is 2. Each unit increase corresponds to 0.5 SD. The test questions are classified as secrecy and were not available to the investigators. The test was created to evaluate the cognitive function of the recruits. The outcome of the test is considered as a valid measure of overall cognitive function and has previously been described in several large cohort studies in a wide range of fields [9, 20–23].

### Statistical Analysis

Data are presented as mean (SD) or numbers and proportions. Descriptive univariate analysis (chi-2) was used to compare characteristics of the study cohort with those of the group with missing data. Ordinal regression analysis was used to calculate the odds ratio (OR) for a cognitive function stanine score above the average of men in a selected reference category in each factor. Men born at term (GA 37–42 weeks), men with average BWSDS (-1 to <+1), and men with average W_max_ (276–325 W) were chosen as references. Crude and adjusted OR´s were calculated. In the adjusted regression models, all co-variates significantly associated with cognitive function were adjusted for in a stepwise manner. Differences with a p-value <0.05 were considered statistically significant. Statistical analyses were performed with Statistica software version 9.0, Statsoft Inc., Tulsa, US and IBM SPSS Statistics Version 23.

## Results

### Population characteristics

During the period 1993 to 2001, 428,092 men conscripted for military service. After exclusion of men not included in the MBR and of men with missing (no data on exercise or cognitive function test) or misclassified data, the final study cohort consisted of 218,802 men (Fig 1). A comparison of all perinatal, parental and conscription characteristics in the study cohort and those who were not included has previously been published [13]. The perinatal characteristics and the results on the cognitive function test of the study cohort and those not included were similar, however, although the differences were small they were all statistically significant (p<0.01). Nonetheless, the most striking difference between the groups was a lower proportion of men with full health (A-status) among those who were not included than in the study cohort (Table 1).

**Table 1:** Description of perinatal and adult characteristics of the study cohort and of those excluded because of missing register data.

	Study cohort N = 218802		Excluded subjects N = 209290	
Gestational Age (GA)	n	%	n	%
<28w	56	0.0	109	0.1
28-31w	726	0.3	684	0.4
32-36w	9927	4.5	8741	5.0
37-41w	182477	83.4	146500	84.0
≥42w	25616	11.7	18329	10.5
Missing*	0		34927	
Total	218802	100.0	209290	100.0
Birth Weight Standard Deviation Score (BWSDS)				
<-2	7051	3.2	5419	3.1
-2 to <-1	35744	16.3	27539	15.9
-1 to <+1	147805	67.6	117811	67.9
+1 to <+2	22970	10.5	18473	10.6
≥+2	5232	2.4	4378	2.5
Missing*	0		35670	
Total	218820	100.0	209290	100.0
Adult Health Status				
A	128079	59.4	45999	24.0
B	14563	6.8	9305	4.8
D	32115	14.9	18979	9.9
E	11208	5.2	16591	8.6
J	7848	3.6	15830	8.2
JC	98	0.0	5498	2.9
Y	20914	9.7	76671	39.9
Z	750	0.3	3139	1.6
Missing*	3227		17278	
Total	218802	100.0	209290	100.0
Adult Cognitive test (stanine score)				
1	47284	21.6	49684	23.7
2	49496	22.6	41038	19.6
3	46972	21.5	56945	27.2
4	41594	19.0	36400	17.4
5	28834	13.2	23894	11.4
≥6	4622	2.1	1288	0.6
Missing*	0		41	
Total	218802	100.0	209290	100.0

* In order to enhance comparability between the study cohort and the excluded subjects, the missing subjects have not been included in the percentage. GA, BWSDS and results from cognitive testing were among the inclusion criteria for the study and therefore have no missing values in the analyzed cohort.

### Preterm birth, adult exercise capacity and cognitive function

The mean stanine score for cognitive function in men born at term with normal birth weight was 2.9 ±1.4 (SD). In crude analyses, men born extremely (GA<28 weeks) and moderately preterm (GA = 32–36 weeks), as well as men born post term (≥42 weeks) exhibited statistically significant lower ORs to achieve a stanine score for cognitive function exceeding 2.9, whereas men born after 28–32 weeks of gestation had similar odds for cognitive function stanine score above 2.9 as men born at term. The OR for having a cognitive function test result above average decreased with decreasing BWSDS and with decreasing W_max_ (Table 2).

**Table 2:** Mean stanine scores and Odds Ratios (OR) for cognitive function >2.9 in relation to Gestational age (GA), Birth Weight Standard Deviation Score (BWSDS) and maximal exercise capacity (W_max_).

*GA*	*N*	*Mean Stanine score (SD)*	*OR (95% CI) for stanine score >2*.*9*		
			*crude*	*model 1*	*model 2*
*<28w*	*56*	*2*.*4 (1*.*4)*	*0*.*51 (0*.*31–0*.81)*	*0*.*57 (0*.*36–0*.*91)*^¤^	*0*.*60 (0*.*38–0*.*97)*^¤^
*28-31w*	*726*	*2*.*9 (1*.*4)*	*0*.*99 (0*.*87–1*.*12)*	*1*.*02 (0*.*90–1*.*16)*	*1*.*04 (0*.*91–1*.*18)*
*32-36w*	*9927*	*2*.*8 (1*.*4)*	*0*.*94 (0*.*91–0*.98)*	*0*.*95 (0*.*92–0*.99)*	*0*.*96 (0*.*93–1*.*00)*^¤^
*37-41w*	*182477*	*2*.*9 (1*.*4)*	*1*.*00*	*1*.*00*	*1*.*00*
*≥42w*	*25616*	*2*.*8 (1*.*4)*	*0*.*96 (0*.*94–0*.*98)*^#^	*0*.*97 (0*.*95–0*.99)*	*0*.*97 (0*.*94–0*.99)*
*BWSDS*					
*-2*	*7051*	*2*.*8 (1*.*4)*	*0*.*91 (0*.*88–0*.*95)*^#^	*0*.*96 (0*.*92–1*.*01)*	*0*.*96 (0*.*92–1*.*01)*
*-2 to <-1*	*35744*	*2*.*8 (1*.*4)*	*0*.*95 (0*.*93–0*.*96)*^#^	*0*.*97 (0*.*95–0*.99)*	*0*.*97 (0*.*95–0*.99)*
*-1 to <+1*	*147805*	*2*.*9 (1*.*4)*	*1*.*00*	*1*.*00*	*1*.*00*
*+1 to <+2*	*22970*	*2*.*9 (1*.*4)*	*1*.*05 (1*.*03–1*.*08)*^#^	*1*.*03 (1*.*01–1*.06)*	*1*.*03 (1*.*01–1*.*06)*^¤^
*≥+2*	*5232*	*2*.*9 (1*.*4)*	*1*.*07 (1*.*02–1*.13)*	*1*.*05 (1*.*00–1*.*10)*^¤^	*1*.*05 (1*.*00–1*.*10)*
*W*_*max*_					
*≤225*	*11726*	*2*.*4 (1*.*3)*	*0*.*57 (0*.*55–0*.*59)*^#^	*0*.*57 (0*.*55–0*.*59)*^#^	*0*.*56 (0*.*54–0*.*58)*^#^
*226–275*	*46171*	*2*.*8 (1*.*4)*	*0*.*92 (0*.*90–0*.*94)*^#^	*0*.*92 (0*.*90–0*.*94)*^#^	*0*.*91 (0*.*90–0*.*93)*^#^
*276–325*	*76384*	*2*.*9 (1*.*4)*	*1*.*00*	*1*.*00*	*1*.*00*
*326–375*	*67896*	*2*.*9 (1*.*4)*	*1*.*06 (1*.*04–1*.*08)*^#^	*1*.*05 (1*.*04–1*.*07)*^#^	*1*.*05 (1*.*03–1*.*07)*^#^
*≥376*	*16625*	*3*.*0 (1*.*4)*	*1*.*13 (1*.*09–1*.*16)*^#^	*1*.*12 (1*.*08–1*.*15)*^#^	*1*.*11 (1*.*08–1*.*14)*^#^

Model 1; OR adjusted for GA, BWSDS and Wmax. Model 2; OR adjusted for GA, BWSDS, Wmax, maternal age, maternal origin of birth, parity, singleton or multiple birth, parental income, parental educational level, socioeconomic index, BMI, blood pressure, health status.

^#^ p<0.001,

* p<0.01,

^¤^ p<0.05

The maternal, perinatal and socioeconomic co-variates listed in the methods section all had a statistically significant association with cognitive function. Accordingly, they were all included in the fully adjusted regression model (Table 2). In the adjusted analyses, the effects of gestational age and BWSDS on later cognition diminished or became insignificant, whereas the association between low physical exercise capacity and low cognition remained essentially unchanged (Table 2).

**Fig 2:**
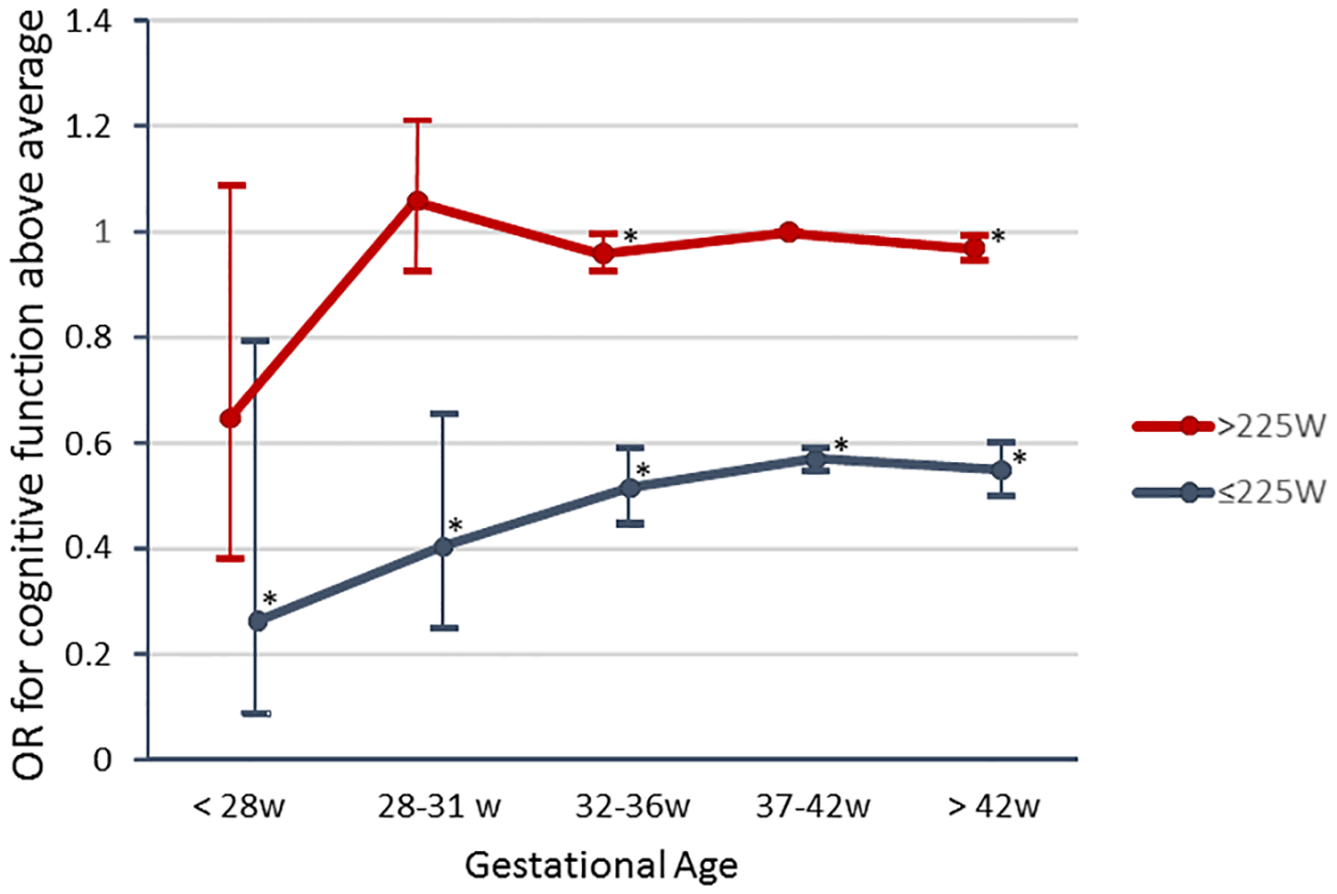
Associations of Odds Ratio (OR) for higher cognitive function with Gestational Age (GA) and maximal exercise capacity (W_max_). Men exhibiting a low exercise capacity had lower ORs for cognitive function (blue line) compared to men with average to high W_max_ (red line). *p<0.05.

The lowest adjusted ORs for having a cognitive function above average was seen in men born extremely preterm (aOR = 0.60; 95%CI: 0.38–0.97) and in men with the lowest exercise capacity, i.e., ≤225W (aOR = 0.57; 95%CI: 0.54–0.58). In men born extremely preterm, the effect on cognition was modified mainly by adjusting the OR for exercise capacity (Table 2). To further explore any effect-modification of exercise capacity on GA, subjects were stratified into low range W_max_ (≤225W) or regular-high range W_max_ (>225W). In Fig 2 the combined effect of GA and W_max_ on ORs for a cognitive function stanine score above average is shown. For each GA, subjects with regular-high physical exercise capacity (>255W) had higher OR for a cognitive function above average. The lowest odds ratio for a cognitive function stanine score above average was seen in young men born extremely preterm (GA <28w) and with a low W_max_ (OR = 0.26; 95%CI:0.09–0.82) whereas extremely preterm men with an exercise capacity in the regular-high range had similar or even higher ORs (OR = 0.59; 95% CI:0.35–0.99) for cognitive function as men born at term with low Wmax (OR = 0.57; 95% CI:0.55–0.59).

## Discussion

This is the first study to show that low exercise capacity is associated with lower cognitive function in young healthy men born preterm. The association remained when the results were adjusted for possibly confounding factors such as fetal growth and educational level of the parents. These results suggest that improving physical fitness may be a means to improve cognitive outcome in children growing up following preterm birth. This hypothesis is supported by the fact that the effect of GA and BW on cognitive function is decreased when adjusting for exercise capacity and the growing number of studies showing that exercise improves cognition function [8–10].

In concordance with several previous studies, our data demonstrate that higher cognitive function is associated with higher exercise capacity [9] and that low cognitive function is associated with lower GA (Table 2). Cognitive function is a complex variable dependent on both genetic and environmental factors. Indeed, the same features that are important to develop a high cognitive function may also be useful when improving exercise capacity, which could explain the association of exercise capacity and intelligence in population-based studies. Nevertheless, there is an increasing amount of evidence illustrating that exercise is good for developing, maintaining and regaining cognitive function [8, 12]. Exercise improves brain plasticity, which means the ability to manage new environments or situations and is an important factor in cognitive function [8]. Moreover, exercise stimulates vessel growth in the nervous system [24] and production of brain-derived neurotrophic factor (BDNF), a factor which is important for synaptic plasticity [25]. In a population-based study, young men improving their exercise capacity between the age of 15 and 18 also exhibited increased cognitive functions over time compared to men not improving their exercise capacity [9], suggesting that improved physical fitness may be beneficial for improving cognitive function in adolescence. Altogether, these reports support the hypothesis that physical exercise may enhance cognitive function. Even though this study doesn’t have any information about how physically active the recruits were, high exercise capacity suggests a higher level of physical activity.

Several observations have confirmed that preterm birth is correlated to a lower cognitive function [4, 9, 26–29]. Similarly, in this study we observed lower cognitive function for men with a gestational age <28 weeks and 32–36 weeks, though no statistical difference was shown for men born with a GA 28–31 weeks. The relatively low number of individuals in this group and the low range of the results on the cognitive function test may have affected the results, as well as the register-based design. The differences in cognitive function between preterm and term subjects were slightly smaller in our cohort compared to previous reports [29], however, given the selected group of healthy survivors this could be expected.

Regardless of GA, low exercise capacity was associated with lower cognitive function (Table 2, Fig 2). As illustrated by our data, the group of men with the combination of low exercise capacity (<225W) and also the lowest GA (<28w) was the group who exhibited the lowest results on the cognitive function test (Fig 2).

Ex-preterm individuals may have a lower exercise capacity due to affected vascular development [30–32] or as an effect of a poor lung function [33]. Furthermore, their ability to or interest in physical activity may be affected, which may lead to a lower exercise capacity. This is further discussed in our previous study [13]. Even if exercise capacity is lower, Clemm and colleagues have shown that individuals born preterm do have the possibility to increase their exercise capacity with increased physical activity [14]. Consequently, increased physical activity may be a possible intervention for children born preterm. Ex-preterm individuals have an increased risk of impaired motor control, both if they are apparently healthy [34] or if they have a known neurological disability [35]. This may theoretically be a reason for lower physical activity and result in lower exercise capacity. Early interventions on motor control could be a possible way to improve the ability for physical activity, which may have positive effects on cognitive function, though more studies are warranted [36] before any conclusion could be drawn.

Poor intrauterine growth has been found to affect cognitive function. Our data revealed small differences that are further decreased when the results are adjusted for exercise capacity, which would probably make them clinically negligible (Table 2). In other respects, our methodology does not allow the analysis of cognitive function in the subgroup with extreme intrauterine growth restricted subjects and no conclusions can be drawn regarding that specific group.

The strengths of this study are its population-based design, the size of the cohort and the possibility to adjust for important covariates and confounders. There are however several limitations that need to be considered when interpreting the results. First, only men were included and the results may therefore not be applicable to women. However, studies that targeted the sex-differences on the effect of exercise on cognitive function have rather shown a greater effect on females than on males [37, 38]. Second, register data were used and it is impossible to know what effort the men put into the exercise test and the cognitive function test. Though the instruction was to put maximal effort into the tests and the circumstances were the same for all men. Third, the actual level of physical activity was never measured in this study. A physically active lifestyle can be assumed to often, but not always improve physical fitness. Subjects may exhibit a physically active behavior but be unable to perform well in exercise capacity tests and assessing physical activity would require other measures, such as accelerometry. During childhood, physical activity in daily life may be even more important for physical fitness and act as a determinant for future health, but that remains to be shown. In our study only aerobic fitness at young adulthood was tested and that has to be considered when interpreting the results. Fourth, the study has a cross-sectional design and causality can therefore never be claimed. Fifth, the men in the cohort were born 1973–1983 and their outcome may not be applicable on today’s preterm survivors. Given the huge improvements in neonatal care, these survivors have to be assumed to have been among the strongest and healthiest, otherwise they would not have survived. The cohort is probably comparable to a selected group of today’s preterm infants with few neonatal complications. The men that performed the exercise test at conscription are even further selected, since good health was a criterion for the test. Probably the differences would be larger in a less selected group of preterm survivors and the association between aerobic fitness and cognitive function is likely to be similar. The mandatory conscription does no longer exist which makes the study impossible to do on a more contemporary cohort.

Furthermore, the test used for cognitive function is not a standardized test. It has been developed by the Swedish military to test cognitive function and the questions are classified as secrecy and thereby not available to the authors. It cannot be used as to give a certain IQ score. Nevertheless, all men performed the same test with the same set of multiple choice questions and it is reasonable to compare individuals amongst each other. The stanine score is a score between 1 and 9 and each unit increase corresponds to an increase of the results on the test of 0.5 SD. Average should then be 5, however, the results that the men in this study achieved were mainly between 1 and 6. Only a few of the men had scores ≥7. Other studies using Conscription data seem to have the same results [9]. One explanation could be the lower effort that the conscripted men put into the test compared to the ones used when developing the test.

Nor was the exercise test a standardized test. In standard maximal tests on cycle ergometer the subjects will cycle two-three minutes on each level compared to one minute in this study [39]. It may have affected the results in a positive way. On the other hand, the test circumstances were the same for all subjects.

We have previously shown that preterm birth is associated with lower exercise capacity [13]. In this study we extend these findings and show that low gestational age in combination with low exercise capacity is associated with the lowest cognitive function. Though, given the limitations of this study, further evidence is necessary to prove this. However, taken together with other observations showing a beneficial effect of exercise on cognitive function, we suggest that improving aerobic fitness may be a way to improve cognitive function in survivors of preterm birth, though further studies are warranted.

## Supporting Information

S1 Table Explanation of codes classifying health status at recruitment. (DOCX)
